# Characterizing Populations Based on Proximity to a Fragile X Syndrome Specialty Clinic

**DOI:** 10.1080/19447515.2026.2662302

**Published:** 2026-05-07

**Authors:** Hidayat Y. Ogunsola, April D. Summers, Maria G. Gonzalez, Angela M. Thompson-Paul, Marc Schweizer, Robert M. Miller, Hilary Rosselot, Sarah C. Tinker

**Affiliations:** a Centers for Disease Control and Prevention, Atlanta, GA; b Oak Ridge Institute for Science and Education, Oak Ridge, TN; c U.S. Public Health Service, Rockville, MD; d National Fragile X Foundation, Washington, DC

**Keywords:** Fragile X syndrome, healthcare access, rare diseases

## Abstract

Fragile X syndrome (FXS) is a rare condition that can require lifelong specialized care for optimal management. We describe census-based characteristics of general U.S. populations defined by proximity to FXS specialty clinics to better understand potential barriers to access. We used Geographic Information System software to estimate drive times from U.S. census tract centroids to FXS clinics. Of the contiguous U.S. population, 36.4% lives ≤1 hour from the closest FXS clinic in the same state; 32.4% live >1 to 4 hours, 7.3% live >4 hours, and 23.8% have no FXS clinic in their state. Individuals residing >1 hr from or in a state without an FXS clinic, compared to <1 hr, were more commonly non-Hispanic White, had household income below 150% poverty level, had one or more disabilities, and lived in a household without an internet subscription. Populations living ≤1 hr from an FXS clinic more commonly spoke limited English, lived in multiunit housing, and did not have a vehicle. These results can be used to identify areas of higher need for improving access to specialty care, inform efforts to reduce disparities related to access, and facilitate more optimal healthcare for people with FXS.

## Résumés en Français

Le syndrome de l’X fragile (SXF) est une condition rare qui peut nécessiter des soins spécialisés tout au long de la vie pour une prise en charge optimale. À l’aide des données issues du recensement aux États-Unis, nous décrivons les caractéristiques des populations en fonction de leur proximité avec des cliniques spécialisées dans le SXF afin de mieux comprendre les obstacles potentiels à l’accès aux soins. Nous avons utilisé un logiciel de SIG pour estimer les temps de trajet en voiture entre les centers des secteurs de recensement américains et les cliniques spécialisées dans le SXF. Parmi la population des États-Unis contigus, 36,4 % vivent à ≤1 heure de la clinique SXF la plus proche dans le même État; 32,4 % vivent à >1 à 4 heures, 7,3 % vivent à >4 heures, et 23,8 % n’ont aucune clinique SXF dans leur État. Les personnes résidant à plus d’une heure d’une clinique SXF ou dans un État sans clinique SXF, par rapport à celles vivant à moins d’une heure, étaient plus souvent des Blancs non hispaniques, avaient un revenu familial inférieur à 150 % du seuil de pauvreté, présentaient un ou plusieurs handicaps et vivaient dans un foyer sans abonnement Internet. Les populations vivant à moins d’une heure d’une clinique spécialisée dans le SXF parlaient plus souvent un anglais limité, vivaient dans des logements multi-unités et ne disposaient pas de véhicule. Ces résultats peuvent être utilisés pour identifier les zones où le besoin d’améliorer l’accès aux soins spécialisés est le plus grand, orienter les efforts visant à réduire les disparités liées à l’accès et faciliter une prise en charge de santé plus optimale pour les personnes présentant un SXF.

## Resumenes en Español

El síndrome del cromosoma X frágil (SXF) es una enfermedad poco frecuente que puede requerir atención especializada de por vida para un manejo óptimo. Describimos las características, basadas en datos del censo, de la población general de los Estados Unidos en función de su proximidad a clínicas especializadas en SXF, con el fin de comprender mejor las posibles barreras de acceso. Utilizamos software SIG para estimar los tiempos de viaje desde los centros de las áreas censales de EE. UU. hasta las clínicas para SXF. De la población de los Estados Unidos continentales, el 36,4 % vive a ≤ 1 hora de la clínica para SXF más cercana en el mismo estado; el 32,4 % vive a entre 1 y 4 horas, el 7,3 % vive a más de 4 horas y el 23,8 % no tiene ninguna clínica para SXF en su estado. Las personas que residen a más de una hora de una clínica de síndrome del cromosoma X frágil o en un estado sin dicha clínica, en comparación con las que residen a menos de una hora, son con mayor frecuencia blancas no hispanas, tienen ingresos familiares inferiores al 150 % del umbral de pobreza, presentan una o más discapacidades y viven en hogares sin acceso a internet. Las poblaciones que viven a una hora o menos de una clínica de SXF suelen tener un dominio limitado del inglés, viven en viviendas multifamiliares y no poseen vehículo propio. Estos resultados pueden utilizarse para identificar áreas con mayor necesidad de mejorar el acceso a la atención especializada, orientar los esfuerzos para reducir las desigualdades relacionadas con el acceso y facilitar una atención médica más óptima para las personas con SXF.

## Introduction

Fragile X syndrome (FXS) is an X-linked dominant inherited neurodevelopmental disorder that affects about one in 7,000 males and about one in 11,000 females (Hagerman et al., 2017; Hunter et al., 2014). FXS is caused by a mutation in the Fragile X messenger ribonucleoprotein 1 (FMR1) gene. The FXS phenotype commonly includes intellectual disability, developmental delay, and connective tissue differences (Hagerman & Hagerman, 2025). Conditions that commonly co-occur in individuals with FXS include autism, attention deficit hyperactivity disorder, social anxiety, and seizures (Raspa et al., 2017). Females with FXS are usually less affected than males because they have another X chromosome to partially compensate for the damaged one (Hagerman et al., 2017).

FXS can require lifelong specialized care for optimal management of symptoms and co-occurring conditions. However, many individuals with FXS face significant healthcare disparities, particularly inadequate access to specialized healthcare (Krahn et al., 2006). In the United States, availability and accessibility of healthcare can be challenging for conditions that are best accommodated by specialized care centers. In a survey of over 600 caregivers of individuals with FXS, almost 40% reported that their child’s primary healthcare provider was not as knowledgeable about FXS as would be needed for optimal care (Wheeler et al., 2019). Delayed access to healthcare services for individuals with FXS can be detrimental to physical and mental health, leading to a poorer quality of life (Wheeler et al., 2019).

In recent years, telehealth has been increasingly adopted as a way of accessing and providing care to patients, particularly those living in rural and underserved areas. However, state-specific licensing requirements (Federation of State Medical Boards, 2025) for physicians and other healthcare providers can serve as a barrier to telemedicine, particularly if the patient and provider are located in different states, although some specialties, such as psychology, have interstate compacts that facilitate use of telehealth (e.g., PSYPACT). In addition, lack of reimbursement for telehealth from commercial insurance, Medicare, and Medicaid insurance plans (which are mostly dependent on individual state policies) poses another barrier to the use of telehealth (Gajarawala & Pelkowski, 2021).

There is a lack of research investigating access to specialized care for individuals with FXS. Geographic Information System (GIS) mapping has been used in healthcare for various purposes, including efforts aimed at describing and better understanding the shifting dynamics of healthcare access (Delmelle et al., 2013). We aimed to understand the characteristics of the U.S. population (not specifically people with FXS) related to access to FXS clinics based on drive time proximity by using census data and ArcGIS mapping software. These findings will inform our understanding of groups likely to have more challenging access to specialized FXS care; the potential impact of interstate telehealth, insurance, and licensing policies on access to care; and groups underrepresented in data from clinic-based registries.

## Methods

### Study Design

We conducted an exploratory ecologic study of socioeconomic and demographic characteristics of the populations in the contiguous United States, not limited to those with FXS, hereafter referred to as U.S. population, based on their proximity to FXS clinics. Hawaii and Alaska were excluded from the analysis because neither state has FXS specialty-care clinics, and traveling to clinics in other states by car is not feasible. For this analysis, census tract data were accessed through the Centers for Disease Control and Prevention and Agency for Toxic Substances and Disease Registry Social Vulnerability Index (SVI) (Centers for Disease Control and Prevention [CDC], 2022). This database was derived from the U.S. Census Bureau’s 5-year estimates (2018–2022) of the America Community Survey (United States Census Bureau, 2024a). To identify the FXS clinics, we used the list available from the National Fragile X Foundation website (National Fragile X Foundation, 2024). No information on FXS diagnosis was available.

**Figure 1.**
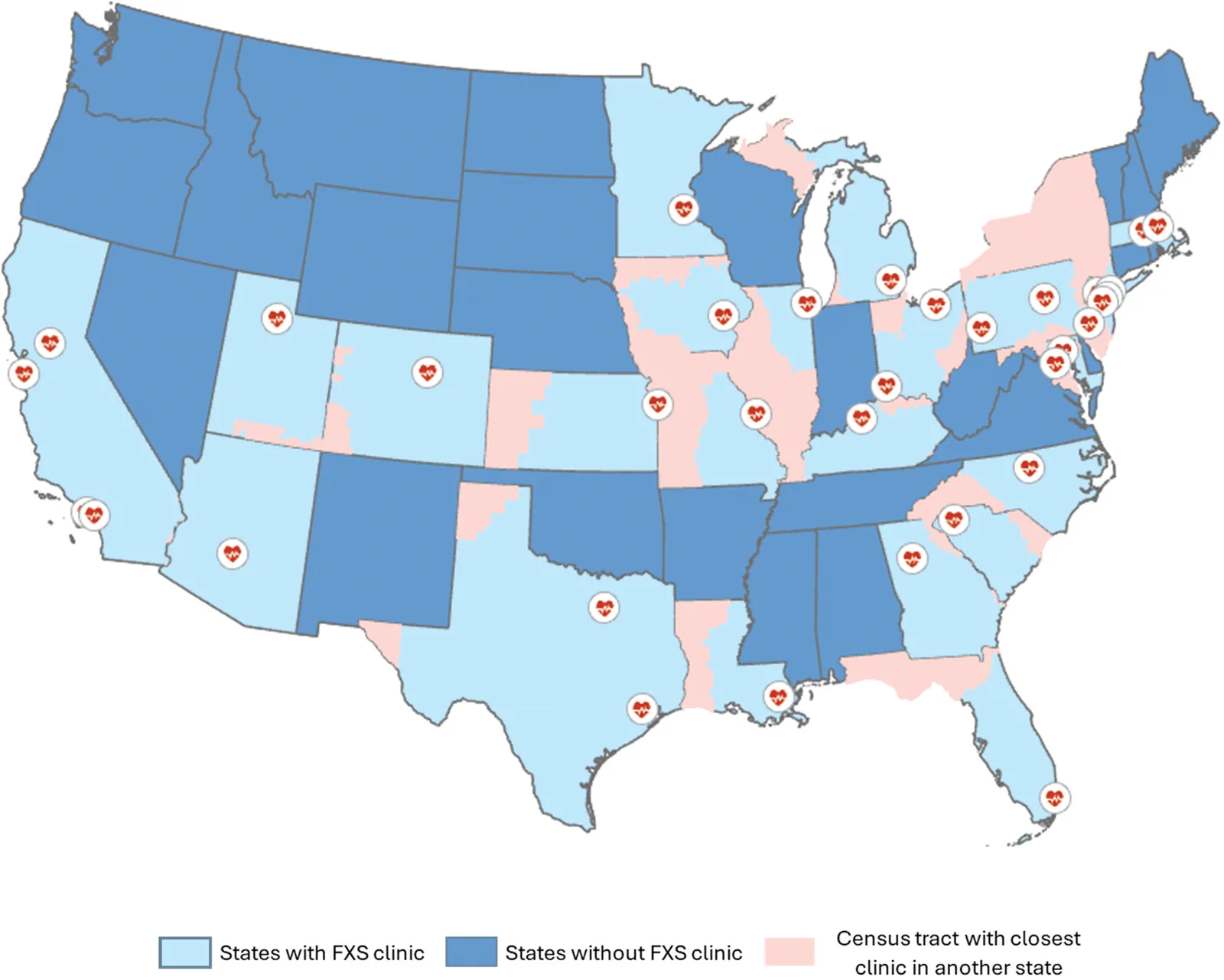
Map of the contiguous United States with geocoded locations of FXS specialty clinics, 2024. *Note*. The pink areas represent census tracts in states with an FXS clinic, but the nearest clinic is in a different state.

As of October 2024, there were 34 FXS clinics in the United States located in 24 states; 7 states had more than one clinic: California (4), Massachusetts (2), New York (2), New Jersey (2), Ohio (2), Pennsylvania (3), and Texas (2) (Figure 1). Among 84,120 census tracts (total population [*N*] = 331,097,593) downloaded from the CDC’s SVI database, we excluded 436 tracts (*n* = 1,450,589) located in Hawaii, 177 tracts ( n= 734,821) located in Alaska, 12 tracts (*n* = 17,453) for which drive times were not generated (e.g., because of location with no nearby roads), and 493 tracts across 45 states with no population data (e.g., because of nonresidential land use). The final number of tracts included in our analysis was 83,002 (total population *N* = 328,894,730).

### GIS Mapping

We used ArcGIS Pro 3.1.7 software to estimate drive times from U.S. census tract centroids to FXS clinics (Pucha-Cofrep & Fries, 2025). The process involved several steps: first, we geocoded FXS specialty clinics onto the U.S. map. Next, we downloaded the United States Census Topologically Integrated Geographic Encoding and Referencing (TIGER/Line) boundary shapefiles (2022) for data visualization at the census tract level (United States Census Bureau, 2024c) and added to the map. To this layer, we joined census tract-level SVI data. We included the following census tract-level census-derived variables in our analysis: race/ethnicity (Hispanic and non-Hispanic American Indian or Alaska Native, Asian, Black, Native Hawaiian or Other Pacific Islander, White, other race, and two or more races), people living below 150% poverty, unemployment (among those 16+ years of age), lack of health insurance, lack of high school diploma (among those 25+ years of age), lack of internet subscription, noninstitutionalized civilian with a disability, people (5+ years of age) who speak English “less than well,” living in a multi-unit structure (10+ units), no available vehicle, and living in group quarters (i.e., not living in a house, apartment, mobile home, or rented room). White race/ethnicity data were downloaded separately from the United States Census Bureau website (United States Census Bureau, 2023) because it was not part of the SVI variables. To delineate the state boundaries (Figure 1), cartographic boundary shapefiles at the state level (2022) (United States Census Bureau, 2024b) were also downloaded from the U.S. Census Bureau website and added to the map.

Using the Esri Network Analyst Extension in ArcGIS Pro 3.1.7, we used the Closest Facility Solver analysis workflow to calculate the drive time from the centroid of each census tract to the FXS clinics. This workflow has three steps:

1. The *make closest facility analysis layer* tool was used to make a closest facility network analysis layer. This helped determine the closest facility to an incident (described below) based on a travel mode (driving time).
2. We used the *add locations* tool to add input features to the network analysis layer. This tool was run twice, once to add the FXS clinics as a “Facilities” sublayer, and the second time to add the census tracts point locations as an “Incidents” sublayer.
3. We used the *solve* tool to generate the drive times from each census tract to the nearest FXS clinic (regardless of state and, separately, limited to those in the same state).

We created a series of maps to display patterns of selected census tract-level characteristics relative to the location of FXS clinics. We considered the three most prevalent characteristics (excluding race/ethnicity)—living below 150% poverty, lacking health insurance, and with a disability—as well as lack of household internet due to its relationship to telehealth.

### Statistical Analysis

We classified census tracts based on (a) whether there was a clinic in the same state and, (b) if there was at least one clinic in the same state, whether a clinic in another state was nearer to the census tract centroid. We also created three drive time categories: ≤1 hour, >1 to 4 hours, and >4 hours, which have been used in previous similar analyses (Pudrith et al., 2024; Salciccioli et al., 2019). Our primary analysis focused on drive time to the closest clinic in the same state, as many insurance providers prioritize payments for facilities in the same state; census tracts in states without a clinic were considered as a separate category. In sensitivity analyses, we also categorized census tracts based on the drive time to the nearest clinic regardless of whether there was a clinic in the same state.

The percentage of individuals in each of the census categories described above, for each of the three drive time categories and those living in states without clinics, was calculated using the formula:

$$\frac{\sum_{i=1}^{N_{x}}\#\;in\;census\;category\;in\;census\;tract_{xi}}{\sum_{i=1}^{N_{x}}\#\;in\;census\;tract_{xi}}$$

where i represents the census tract and x represents the drive time categories (≤1 hour, >1 to 4 hours, >4 hours, no clinic in state).

Summary statistics were calculated using SAS software v. 9.4 for Windows (SAS Institute Inc., Cary, NC, USA). Statistical testing was not done because the data are not a sample from a population but rather a complete census.

## Results

Approximately three-quarters (76.2%) of the U.S. population lives in a state that has at least one FXS clinic; the closest FXS clinic is within the same state for most people living in a state with an FXS clinic (89.9%). About one-third of the U.S. population lives ≤1 hour from the closest FXS clinic in the same state (36.4%); 32.4% live >1 to 4 hours and 7.3% live >4 hours (Supplementary Table 1). Figure 1 shows the location of FXS clinics and highlights census tracts based on the relative location of clinics in the same state and nearest clinics in another state. The largest density of FXS clinics is in the Northeastern Census Region. A lack of clinics was observed in the northern Pacific, Mountain, West North Central, and East South Central Census Divisions.

### Demographic Characteristics of Populations Based on Drive Times to FXS Clinics

**Figure 2.**
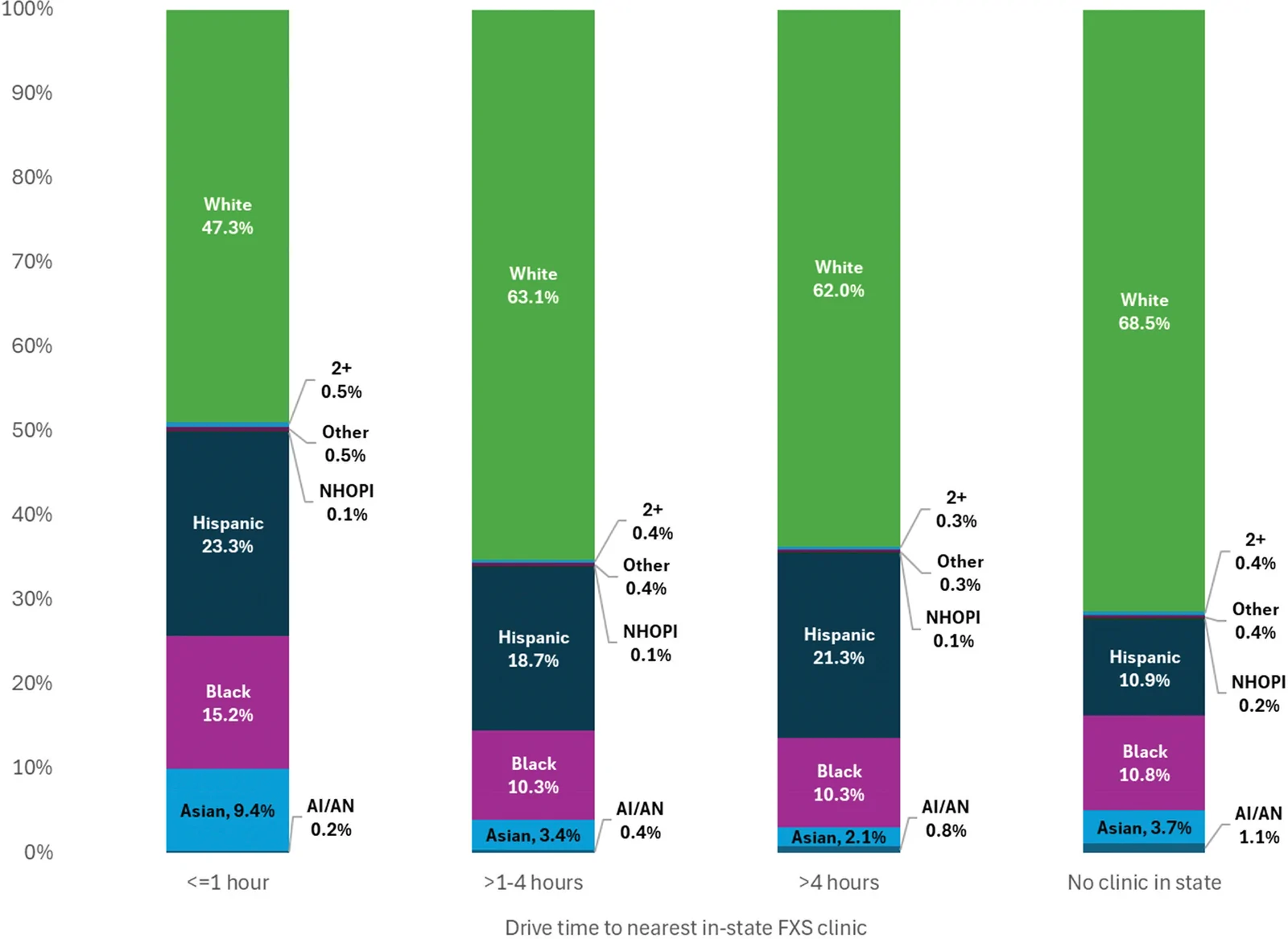
Distribution of race and ethnicity of U.S. population* based on category of drive time to nearest fragile X syndrome (FXS) specialty clinic. *Note.* AI/AN = American Indian / Alaskan Native, NHOPI = Native Hawaiian and Other Pacific Islander *Population in contiguous United States; Drive time categories are based on the shortest drive time from a census tract to a clinic located in the same state All race categories are non-Hispanic; 2+ indicates two or more races.

Several differences in the racial/ethnic distribution of the populations in each of the drive time categories were observed (Figure 2; Supplementary Table 1). The percentage of the population identified as American Indian/Alaska Native (AI/AN) increased with increasing drive time category in states with an FXS clinic. Additionally, a greater percentage of the population in states without an FXS clinic were AI/AN (1.1%) compared to states with an FXS clinic (0.2 to 0.8%). There was a higher percentage of Asian and Black populations living ≤1 hour from an FXS clinic compared to those living more than 1 hour away or in a state without an FXS clinic. Hispanic individuals comprised 23.3% of the population living ≤1 hour and 21.3% of the population living >4 hours from the nearest clinic in their state; they comprised only 10.9% of the population living in a state without an FXS clinic. Approximately two-thirds of the population living ≥ 1 hour from the closest FXS clinic in their state and in a state without an FXS clinic were White, while only slightly less than half of the population living ≤1 hour from the closest FXS clinic were White. Finally, the percentages of the population that were Native Hawaiian or Other Pacific Islander, other race, and two or more races were similar across the four drive time categories.

### Socioeconomic and Household Characteristics of Populations Based on Drive Times to FXS Clinics

**Figure 3.**
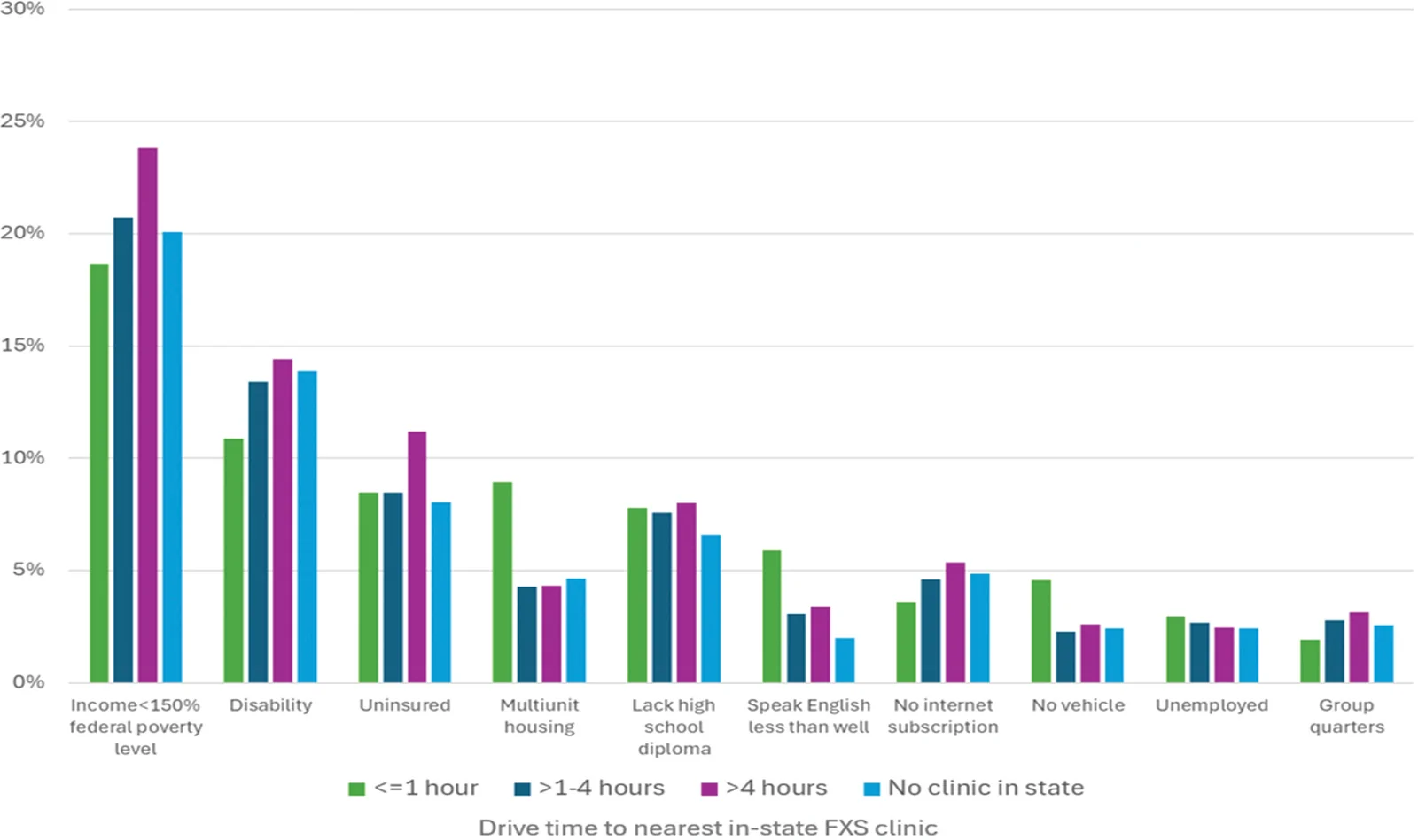
Distribution of selected characteristics of U.S. population* based on category of drive time to nearest fragile X syndrome (FXS) specialty clinic *Note.* *Population of contiguous United States. Drive time categories are based on the shortest drive time from a census tract to a clinic located in the same state. Unemployment is among civilians aged ≥16 years of age. Lack of high school diploma is among those ≥25 years of age. Disability status is among the civilian noninstitutionalized population. Speak English less than well is among those ≥5 years of age. Multiunit housing is defined as structures with 10 or more units.

The percentage of the population with several of the SVI characteristics increased with increasing drive time to the closest in-state FXS clinic (≤1 hr to >4 hr, respectively), specifically income below 150% of the poverty level (18.6% to 23.8%), with a disability (10.9% to 14.4%), without an internet subscription (3.6% to 5.4%), and living in group quarters (1.9% to 3.1%) (Figure 3; Supplemental Table 1). The percentage of individuals who were unemployed slightly decreased with increasing drive time (3.0% to 2.5%). The population living >4 hours from an FXS clinic had a higher percentage lacking insurance (11.2%) than the populations living closer (8.5% in each) and in states without an FXS clinic (8.0%). Having no high school diploma was more common among individuals residing in states with an FXS clinic, regardless of drive time (7.6% to 8.0%), compared to states without an FXS clinic (6.6%). Characteristics more prevalent for the population living ≤1 hour from an FXS clinic than the populations living 1 to 4 hours, >4 hours, or in a state without an FXS clinic, respectively, were living in multiunit housing (8.9% versus 4.3% to 4.6%), speaking English less than well (5.9% versus 2.0% to 3.4%), and lacking a vehicle (4.6% versus 2.3% to 2.6%). When we considered drive time categories not requiring FXS clinics be in the same state as the census tract (Supplementary Table 2), results were similar.

### Map Patterns

**Figure 4.**
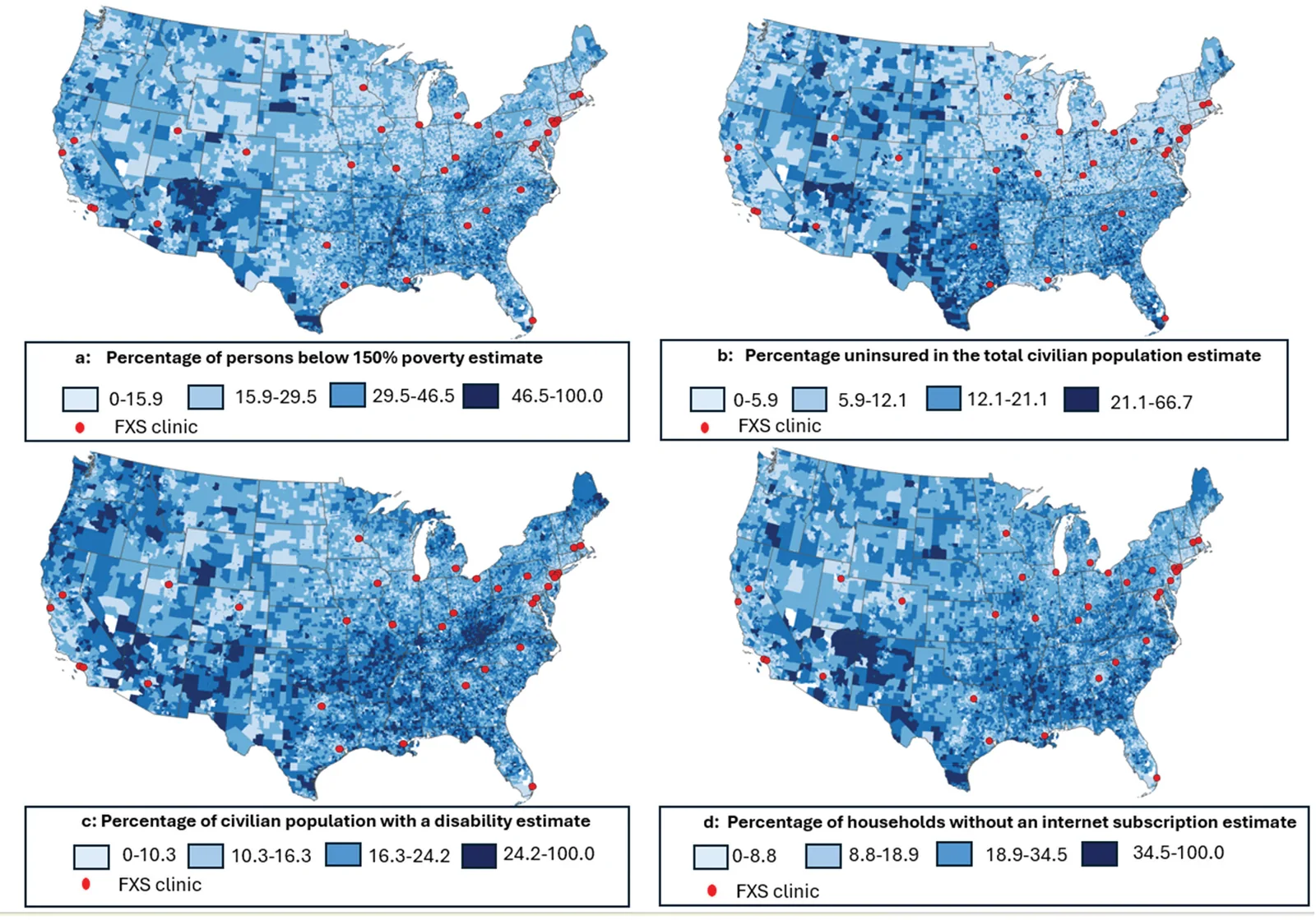
Distribution of selected census tract-level characteristics in relation to the location of fragile X syndrome (FXS) specialty clinics.

The map depicting the distribution of individuals living below 150% poverty level (Figure 4(a)) shows higher concentrations of poverty in the Southeast and Southwest regions of the country, areas with a lower density of FXS clinics. The map depicting the distribution of lack of insurance coverage (Figure 4(b)) shows that, in general, many areas with lower insurance coverage also have a lower density of FXS clinics. However, in some areas with one or more FXS clinics, higher percentages of the population are uninsured. In addition, in some instances, insurance patterns follow state lines, a different pattern than observed for the other characteristics considered. Overall, higher percentages were uninsured in the West South Central, South Atlantic, and Mountain divisions. The map showing the distribution of noninstitutionalized civilians with a disability (Figure 4(c)) shows higher percentages across the Upper South/Appalachia, Southwest, and Northwest regions, which have fewer FXS clinics. The map displaying the distribution of households lacking an internet subscription (Figure 4(d)) shows “halos” of higher percentages of the population with an internet subscription around FXS clinics. Conversely, the Southwest and East South Central through the South Atlantic regions, which largely lack FXS clinics, show lower percentages with an internet subscription.

## Discussion

Approximately three out of four people living in the contiguous United States reside in a state with at least one FXS clinic; however, less than 40% live ≤1 hour drive time of any FXS clinic and only one in three live ≤1 hour drive time to the nearest FXS clinic in their state. Compared to individuals living ≤1 hour drive time of an in-state FXS clinic, those residing farther from FXS specialty care were more commonly non-Hispanic White, had household income below 150% poverty level, had one or more disabilities, and lived in a household without an internet subscription.

While it is important for healthcare providers to have a basic understanding of FXS to facilitate diagnosis and local support, widespread specialized experience is impractical given the low prevalence of FXS in the population. Lifelong, highly specialized care is often needed to optimally support people with FXS, which FXS specialty clinics are well equipped to provide. Increased drive time for specialty care has been associated with worse health outcomes for other rare disorders, including cystic fibrosis and congenital heart defects (Kaltman et al., 2020; Roberts et al., 2016).

An additional issue impacting access to specialized care is insurance coverage. In our study, the population with the longest drive time to an FXS clinic in their state also had the highest percentage without insurance. Even among those with insurance, policies may not cover a visit to a specialist in a different state, even if that provider is closer to or provides specialized care unavailable in the individual’s state. Our data show that 10% of the population would have to travel further to reach a clinic in their state than one in a neighboring state.

Telehealth offers an opportunity for remote specialized care. However, access may still be limited by the same interstate insurance restrictions as in-person visits, and additionally by interstate medical licensure policies. Although specific regulations vary by state and constantly evolve, many states require that telehealth be provided by clinicians licensed in the state where the person is receiving care (Center for Connected Health Policy, 2024), such that even for telehealthcare, patients may have to travel to a different state. The U.S. Centers for Medicare and Medicaid Services provide guidance on coordinating care provided by out-of-state providers for children with medically complex conditions (Centers for Medicare & Medicaid Services, 2021), but specific policy decisions are made by individual states. While many states participate in limited exceptions and reciprocity compacts (Center for Connected Health Policy, 2024), and there are specialties, such as psychology and speech language pathology, with highly adopted telehealth permissive interstate compacts, these regulations are likely to impact access to telehealth for many people with FXS and other conditions requiring specialty care living in states without specialty clinics. A review of 35 randomized-controlled trials funded by the Patient-Centered Outcomes Research Institute assessing various aspects of telehealth identified the need for multisite credentialing as a system-level barrier to telehealth adoption, while partnering with payers to expand insurance coverage was a facilitator (Bailey et al., 2021).

Telehealth services can also be limited by lack of household internet access. Although the percentage of households without an internet subscription was low (<6%) across drive time categories, it was highest for those living furthest from an FXS clinic in their state. The patterns we observed in lack of household internet subscription largely mirror populations living in areas without broadband internet access (Federal Communications Commission, 2025), with fewer subscriptions in more rural areas. Lack of broadband access has been associated with less telehealth use (Zhang et al., 2024), particularly in rural areas (Graves et al., 2021). Although access to telehealth may be provided through cellular networks, rural areas tend to have lower coverage and slower speeds than suburban and urban areas (Federal Communications Commission, 2024). Expanding broadband and cellular infrastructure in these underserved areas could improve specialty healthcare accessibility and support, including for individuals with FXS.

The Fragile X Clinical and Research Consortium (FXCRC) works to promote the establishment of FXS specialty clinics and provides collaborative support among member facilities (Liu et al., 2016). Networks such as FXCRC can amplify the availability of specialized care for rare conditions by sharing best practices, consulting to address specific challenges, and conducting research to improve quality of life. Although specialists may be able to provide consultation to local clinicians less familiar with FXS, participation in clinical trials and receipt of other types of specialized care are unlikely to occur outside of an FXS specialty clinic. However, clinic consortia such as FXCRC are limited in their ability to address many issues related to access, such as state-based reimbursement and licensure regulations and internet availability.

Our results demonstrate the utility of using GIS mapping to characterize populations that may be underrepresented in other data sources. There is currently no population-based surveillance for FXS in the United States, and the low prevalence of the condition precludes use of national surveys to estimate prevalence. Clinic-based data collection efforts have been used to understand better the population with FXS. The largest of these efforts to date is the Fragile X Online Registry with Accessible Research Database (FORWARD), which has data available starting from 2011 and continuing through the present (Sherman et al., 2017). However, a concern with clinic-based studies is that people with access to specialty clinics are unlikely to be representative of all people with that condition.

Compared to overall estimates of the U.S. population in our analysis, there are proportionally more non-Hispanic White participants in FORWARD data (59% and 76%, respectively) and proportionally fewer non-Hispanic Black and Hispanic participants (7% and 13%, respectively) compared to population estimates from our analysis (12% and 19%, respectively) (Choo et al., 2022). These data suggest that issues other than proximal access to FXS clinics are likely to explain underrepresentation of racial/ethnic minority groups in FORWARD.

FXS specialty clinics are generally located in more urban areas, and our data reflected this pattern, with the population living ≤1 hour drive time having characteristics of more urban populations. Urban areas tend to disproportionately concentrate both high-income and low-income households relative to suburban and rural areas (Hegerty, 2023). Almost one in 20 people living ≤1 hour from an FXS specialty clinic did not have access to a vehicle in their household and approximately one in 17 did not speak English well, both of which could impact their ability to access even proximal specialty care. Lack of access to a car and other transportation challenges have been associated with barriers to healthcare in urban youth (Yang et al., 2006), while other studies have shown lack of public transit in more rural areas as a barrier to healthcare access (Sommerhalter et al., 2017). Although the highest percentage of the population living below 150% of the poverty level were those living >4 hours from the closest FXS clinic, almost one in five people living ≤1 hour were in this category. Caregivers of people with FXS with lower family incomes report greater challenges accessing healthcare (Wheeler et al., 2019). Other caregiver-reported reasons for children with FXS not attending a specialty clinic include lack of awareness of clinics or the benefits of attending (Kidd et al., 2017), the frequency of which could differ by demographic characteristics.

Although our results provide population-level information on drive time to the nearest FXS specialty clinic, the interpretation of these results are subject to several limitations. First, we could not quantify the drive time specifically for individuals who need FXS specialty care; results assume similar geographic distribution of individuals with FXS as the general population distribution in the United States. Second, we could not measure other important factors, such as transportation access and caregiver’s ability to take time off of work to attend appointments. Third, FXS specialty clinic locations were based on information provided by the National Fragile X Foundation as of October 2024; there may be other clinics or specialty providers who provide similar services that are not captured by this list. In addition, we did not attempt to quantify or describe availability of and time to FXS clinics using public transportation or differences in level of specialization, quality of care, or types of services provided by the clinics included in this analysis.

Despite these limitations, this analysis provides population-level insights into demographic characteristics potentially related to access to FXS specialty care, based on proximity to current specialty clinics, and may also inform potential biases in participation within clinic-based studies of FXS. These results can identify areas of higher need for providing specialty care and engagement with existing providers. They can also inform the design of future studies and targeted outreach efforts to improve the generalizability of study results. Ultimately, these data can inform efforts to reduce disparities related to access and facilitate more optimal healthcare for people with FXS.

## Supplementary Material

SUP - Ogunsola - Characterizing Populations Based on Proximity to a Fragile X Syndrome Specialty Clinic

Supplemental data for this article can be accessed online at https://doi.org/10.1080/19447515.2026.2662302.
