# Supplementary material for: Characterizing Populations Based on Proximity to a Fragile X Syndrome Specialty Clinic
Source: Am J Intellect Dev Disabil. Author manuscript; Available in PMC 2026 Aug 18. (PMC13479729; doi:10.1080/19447515.2026.2662302)
Supplement: Supplementary file 1 [file NIHMS2198112-supplement-SUP_-_Ogunsola_-_Characterizing_Populations_Based_on_Proximity_to_a_Fragile_X_Syndrome_Specialty_Clinic.docx]

Supplementary Table 1: Distribution of race and ethnicity and other characteristics of U.S. population* based on category of drive time^1^ from census tract to nearest fragile X syndrome (FXS) specialty clinic in the same state

|  | Population living ≤1 hour drive from an FXS specialty clinic | Population living >1 to 4 hour drive from an FXS specialty clinic | Population living >4 hour drive from an FXS specialty clinic | No clinic in state |
| --- | --- | --- | --- | --- |
| Total census tracts | 29,257 | 27,444 | 6,529 | 19,772 |
| Total population percentage^2^ | 36.4% | 32.4% | 7.3% | 23.8% |
| Race/ethnicity^3^ (%^4^) | | | | |
| American Indian or Alaska Native | 0.2% | 0.4% | 0.8% | 1.1% |
| Asian | 9.4% | 3.4% | 2.1% | 3.7% |
| Black | 15.2% | 10.3% | 10.3% | 10.8% |
| Hispanic | 23.3% | 18.7% | 21.3% | 10.9% |
| Native Hawaiian or Other Pacific Islander | 0.1% | 0.1% | 0.1% | 0.2% |
| Other race | 0.5% | 0.4% | 0.3% | 0.4% |
| Two or more races | 3.3% | 3.3% | 3.0% | 3.9% |
| White | 47.3% | 63.1% | 62.0% | 68.2% |
| Socioeconomic status (%^4^) | | | | |
| Below 150% poverty | 18.6% | 20.7% | 23.8% | 20.1% |
| Unemployed^5^ | 3.0% | 2.7% | 2.5% | 2.4% |
| Uninsured | 8.5% | 8.5% | 11.2% | 8.0% |
| No high school diploma^6^ | 7.8% | 7.6% | 8.0% | 6.6% |
| Household characteristics (%^4^) | | | | |
| Households without an internet subscription | 3.6% | 4.6% | 5.4% | 4.9% |
| With a disability^7^ | 10.9% | 13.4% | 14.4% | 13.9% |
| Speak English "less than well"^8^ | 5.9% | 3.1% | 3.4% | 2.0% |
| Housing type/transportation (%^4^) | | | | |
| Multiunit housing^9^ | 8.9% | 4.3% | 4.3% | 4.6% |
| Households with no vehicle available | 4.6% | 2.3% | 2.6% | 2.4% |
| Living in group quarters | 1.9% | 2.8% | 3.1% | 2.6% |

^*^Population in contiguous United States

^1^ Drive time categories are based on the shortest drive time to a clinic located in the same state

^2^ Row percentages

^3^ All race categories are non-Hispanic

^4^ Column percentages

^5^ Unemployment is among civilians aged ≥16 years of age

^6^ Lack of high school diploma is among those ≥25 years of age

^7^ Disability status is among the civilian noninstitutionalized population

^8^ Speak English less than well is among those ≥5 years of age

^9^ Multiunit housing is defined as structures with 10 or more units

Supplementary Table 2: Distribution of race and ethnicity and other characteristics of U.S. population* based on category of drive time from census tract to nearest fragile X syndrome (FXS) specialty clinic^1^ in any state

|  | Population living ≤1 hour drive from an FXS specialty clinic | Population living >1 to 4 hour drive from an FXS specialty clinic | Population living >4 hour drive from an FXS specialty clinic |
| --- | --- | --- | --- |
| Total census tracts | 31,330 | 40,114 | 11,558 |
| Total population percentage^2^ | 39.3% | 47.2% | 13.5% |
| Race/ethnicity^3^ (%^4^) | | | |
| American Indian or Alaska Native | 0.2% | 0.4% | 1.7% |
| Asian | 9.1% | 3.1% | 3.7% |
| Black | 15.3% | 10.6% | 8.8% |
| Hispanic | 22.6% | 15.1% | 20.0% |
| Native Hawaiian or Other Pacific Islander | 0.1% | 0.1% | 0.2% |
| Other race | 0.5% | 0.4% | 0.4% |
| Two or more races | 3.4% | 3.3% | 3.9% |
| White | 48.2% | 66.4% | 61.0% |
| Socioeconomic status (%^4^) | | | |
| Below 150% poverty | 18.5% | 20.6% | 22.5% |
| Unemployed^5^ | 2.9% | 2.5% | 2.5% |
| Uninsured | 8.4% | 8.2% | 10.3% |
| No high school diploma^6^ | 7.6% | 7.3% | 7.5% |
| Household characteristics (%^4^) | | | |
| Households without an internet subscription | 3.6% | 4.8% | 5.0% |
| With a disability^7^ | 10.9% | 13.6% | 14.3% |
| Speak English "less than well"^8^ | 5.7% | 2.5% | 3.2% |
| Housing type/transportation (%^4^) | | | |
| Multiunit housing^9^ | 8.8% | 4.2% | 4.8% |
| Households with no vehicle available | 4.4% | 2.3% | 2.4% |
| Living in group quarters | 1.9% | 2.9% | 2.5% |

^*^Population in contiguous United States

^1^ Drive time categories are based on the shortest drive time to a clinic regardless of whether it is located in the same state

^2^ Row percentages

^3^ All race categories are non-Hispanic

^4^ Column percentages

^5^ Unemployment is among civilians aged ≥16 years of age

^6^ Lack of high school diploma is among those ≥25 years of age

^7^ Disability status is among the civilian noninstitutionalized population

^8^ Speak English less than well is among those ≥5 years of age

^9^ Multiunit housing is defined as structures with 10 or more units
